# REACH Worker Exposure Model for Co-formulants Used in Plant Protection Products

**DOI:** 10.1093/annweh/wxy088

**Published:** 2018-10-31

**Authors:** Volker Mostert, Sebastien Bonifay, Christopher Dobe, Ralph Fliege, Joachim Krass, Renate Vosswinkel, Matthias Wormuth

**Affiliations:** 1 Extera, Langenfeld, Germany; 2 DuPont de Nemours, Mechelen, Belgium; 3 Syngenta Crop Protection AG, Basel, Switzerland; 4 Bayer AG, Crop Science Division, Monheim, Germany; 5 BASF SE, Ludwigshafen, Germany

**Keywords:** co-formulant, exposure assessment, plant protection product, REACH

## Abstract

**Background:**

Substances used as co-formulants in plant protection products (PPP) may require registration under Regulation (EC) No. 1907/2006 (REACH), and additionally where an exposure assessment is required, this must take into consideration the specifics of the PPP use.

**Objectives:**

This work reports a customized screening level model developed to support human health risk assessment of operators, workers, and bystanders (OWB) for co-formulants used in PPP. The OWB model was designed to closely integrate with REACH generic exposure scenarios (GES) for PPP developed by the European Crop Protection Association (ECPA). The use of these tools in combination is expected to lead to a more standardized and hence efficient risk assessment of co-formulants. This study describes the basis for OWB exposure predictions as well as benchmarking against relevant REACH exposure models for equivalent tasks. The benchmarking was carried out to gain some insight into the initial assumption that the most commonly used tier 1 REACH model would be more conservative than the specific PPP models used for regulatory risk assessments under PPP legislation.

**Method:**

Existing exposure models with regulatory acceptance for the most common types of PPP and their professional and consumer uses were selected. The German BBA model was used to assess spray applications. Granule and seed dispersal was assessed using the US Environmental Protection Agency (EPA) Pesticide Handlers Exposure Database (PHED). ECETOC TRA was employed to assess exposure during certain tasks performed in seed treatment, not covered by these PPP models. Where the underlying models featured multiple exposure determinants, the exposure was calculated for all permutations, and the worst-case exposure selected and reported for use in risk assessment. The PPP models are based on measured data collected during actual application of PPP; hence, the worst-case exposure predicted was expected to reflect a realistic worst case for these tasks.

**Results:**

OWB was implemented as an Excel spreadsheet. Exposure models, parameters, and exposure and risk estimates are reported in a REACH-compliant output format to facilitate the registration of co-formulant uses. As would be expected, benchmarking OWB against the PPP-specific exposure models demonstrated equivalence with the worst-case prediction from these underlying PPP models. For the scenarios modelled, the tier 1 ECETOC TRA gave more conservative predictions than OWB. The reduction in conservatism is attributed to the underlying PPP models being based on measured data collected specifically during the use of PPP, compared to the data underlying ECETOC TRA, based mainly on industrial workplace uses.

**Conclusions:**

OWB provides inhalation and dermal exposure estimates for co-formulants used in PPP which are equivalent to the worst-case estimates from existing specialized PPP exposure models based on measured data. OWB has simplified information requirements in comparison to higher-tier REACH or PPP models. Use of OWB in combination with the defined ECPA GES facilitates an efficient and standardized REACH risk assessment and registration of co-formulant uses in PPP. A defined assessment framework and default inputs potentially decreases the anticipated inter-user variability compared with the use of higher-tier PPP or REACH models in this screening level context.

## Introduction

In order to comply with the requirements of Regulation (EC) No. 1907/2006 (REACH), it is necessary to submit a chemical safety report (CSR) for substances manufactured or imported into the European Economic Area at annual quantities greater than 10 tonnes. For substances classified as hazardous or which have persistent, bioaccumulative, and toxic properties, this CSR must include a quantitative exposure assessment and risk characterization, covering all relevant uses.

The European Crop Protection Association (ECPA) has developed standardized generic exposure scenarios (GES), which enable manufacturers and downstream users of substances used as co-formulants in plant protection products (PPP) to readily assess their use in an efficient manner (Dobe *et al*., 2017). To minimize the number of GES and support efficient supply chain communication, a worker exposure model with a suitably broad applicability domain was required to provide conservative exposure predictions for these GES. This study describes the screening level (tier 1) ECPA operator, worker, and bystander model (OWB) which was specifically developed to integrate with these GES.

Co-formulants comprise all ingredients of a PPP other than the active substance (e.g. solvents, emulsifiers, thickeners, colourants, solid carriers, etc.) and are essential for the technical effectiveness of a PPP. Co-formulants need to be registered under REACH, unless exempt, and for hazardous co-formulants, the CSR must include an exposure and risk assessment of the identified PPP uses. No European models are available which have been explicitly developed for the exposure assessment of co-formulants in PPP. The US EPA’s model PIRAT (Pesticide Inert Risk Assessment Tool) appears to be no longer available (Williams *et al*., 2010).

Evaluated and recently validated models are available for the assessment of worker dermal and inhalation exposure under REACH (Lamb *et al*., 2015; Lamb *et al*., 2017; Marquart *et al*., 2017; Tischer *et al*., 2017; van Tongeren *et al*., 2017). ECETOC TRA (ECETOC, 2009; ECETOC, 2012; ECETOC, 2014; ECETOC, 2018) is the model which has been used most frequently for REACH tier 1 exposure assessments, with most of the remaining alternatives classed as higher tier. It provides estimates for dermal and inhalation exposure, and covers tasks potentially related to the use of PPP, such as transfer of solids/liquids and non-industrial spraying. However, these estimates were originally based on data derived from industrial workplace exposure measurements forming the basis of the EASE model (Cherrie *et al*., 2003). Rather than on an exposure-based reason, ECETOC TRA formally places exposure assessment of PPP outside of its applicability domain on the basis of regulatory scope, and concerns about the potential hazard profile of active substances. The recent validation of the ECETOC TRA dermal model included a limited data set collected on PPP (Marquart *et al*., 2017). One of the original assumptions leading to the development of OWB was that the use of tier 1 REACH models (which should be highly conservative) could lead to overestimation of exposure to co-formulants, which depending on the substance hazard profile, could ultimately translate into excessive risk management measures (RMM) stipulated by the manufacturer. A recent evaluation by Spinazzè (2017) of ECETOC TRA for the (out-of-scope) inhalation assessment of sprayed PPP active substances lends weight to this assumption. None of the above REACH models predicts potential worker exposure arising from re-entry of treated fields, and potential exposure of bystanders (general population) to spray drift, which are both standard considerations in agrochemical exposure assessments. It should also be noted that ECETOC TRA inhalation exposure estimates are for vapours and not aerosols.

The metals (EUROMETAUX, 2010), surfactants (AISE, 2010), and solvents (Zaleski *et al*., 2014) industries have also produced specialized exposure models tailored to their specific exposure and use patterns and facilitating sector-specific exposure assessments under REACH.

Several models are available for the regulatory assessment of worker (‘operators’) exposure to active ingredients in PPP. The most important models used in Europe were the ‘German BBA model’ (Lundehn *et al*., 1992) and the UK Predictive Operator Exposure Model (POEM; JMB, 1986). In principle, these can be used for higher-tier risk assessments under REACH, but in practise are unsuitable for screening level assessments due to the number and detail of their exposure determinants. The models are oriented around the assessment of a specific formulation type (CropLife, 2008), with a defined application method, and active ingredient use rate. In contrast, a screening level REACH co-formulant risk assessment must assume that the substance can be used in virtually any formulation type, applied by any method, and at any rate (within reason), up to 8 h/day. The models also use an internal dose as reference for risk assessment—the acceptable operator exposure level (AOEL), rather than the external dose—derived no effect level (DNEL) used under REACH, and thus require a value for dermal absorption (assumptions on which are inherently included in the DNEL_dermal_). All of these aspects reduce the likelihood of their successful and regular use in REACH exposure assessments.

The EFSA has recently released a new harmonized and integrated model (AOEM) for the exposure assessment of workers, bystanders, and residents to PPP (EFSA, 2014). Because it was not available at the time OWB was developed, it is not considered in detail here. However, the same issues described for the previous PPP exposure models prevent its simple use in the screening level assessments for which OWB was envisaged: an increased number of inputs (seven exposure determinants each with between 1 and 21 possible values), and use of differently derived reference doses. The significance of inter-user variability in even relatively simple exposure assessments has been clearly demonstrated (Schinkel *et al*., 2014; Riedmann *et al*., 2015; Money *et al*., 2016; Savic *et al*., 2016; Lamb *et al*., 2017). This combined with the AOEM input information requirements, highly variable exposure assessments by co-formulant manufacturers could be anticipated in practise, even if used in conjunction with the ECPA GES.

This study describes the assumptions and models embedded in OWB as used to date in REACH registrations, and the resulting applicability domain focusing on professional uses. Future updates to OWB are planned to replace some of the underlying models, such as AOEM, using a similar approach. The benchmarking is intended purely to give an indication of the relative predictions of ECETOC TRA, should it have been used by registrants, compared to OWB and the worst-case predictions from selected PPP regulatory models. Further details on the adapted models are provided in the Supplementary Material (available at *Annals of Work Exposures and Health* online), including assumptions and parameters values.

## Methods

### Model selection

The criteria for model selection for use in OWB were regulatory acceptance within European PPP authorizations, non-proprietary and open access to the model data/algorithms, ease of implementation in Excel, and alignment with the task-based assessment approach used under REACH.

Where exposure predictions for several application methods were available in a PPP model, the exposure was calculated for all options, and the worst case selected as the output for risk assessment. Similarly, for a given exposure determinant in a model, exposure estimates were calculated for all possible variants, and the worst-case predictions selected. This was done to be fully transparent and reflect the difficulty in predicting the worst-case exposure when different combinations of PPE are selected across correlated contributing scenarios. The combination of the underlying models and their range of exposure determinants effectively define the applicability domain of OWB.

### Software implementation

OWB (version 3.3) is implemented in Microsoft Excel 2003, and the tool is freely available for download via the ECPA webpage (ECPA, 2015).

The OWB tool requires substance-specific inputs: vapour pressure (Pa), intrinsic physical state of the substance (solid, liquid), DNELs for inhalation (mg/m^3^), and dermal (mg/kg body weight (bw) or mg/cm^2^) exposure. The substance application rate (kg/ha), type of personal protective equipment (gloves, coveralls, respiratory protection), and for seed treatment the substance concentration and use of local exhaust ventilation are inputs specifying the operational conditions (OC) and RMM.

The user can either define the substance application rate for each scenario, or let the tool maximize the use rate for each scenario against a specified target risk characterization ratio (RCR), defined as the exposure divided by the DNEL. The maximization routine explicitly takes into account the correlation between contributing scenarios, i.e. where relevant it is assumed that the same workers conduct mixing and loading, as well as apply the products.

### Identified uses and use descriptor assignment

A screening level REACH risk assessment of a co-formulant must cover the typical activities involving the handling of PPP: mixing, loading, and spraying of PPP; treating of seeds with PPP; and the sowing of treated seeds and dispersal of granular PPP to soil. These activities have been translated into REACH use descriptors (ECHA, 2010), and standardized in GES for professional workers (farmers) and consumers (‘amateurs’) in Table 1. The rationale behind the use mapping, use descriptor assignment, and GES development has been described elsewhere (Dobe *et al*., 2017). OWB was explicitly developed to provide the conservative dermal and inhalation exposure estimates required for each contributing scenario listed in Table 1. The applicability domain/scope of OWB for each contributing scenario (given in column 5) is defined by the selection of the underlying models, and further details are provided in the following sections.

**Table 1.** Generic exposure scenarios described by Dobe *et al*. (2017) for co-formulants in PPP and the use descriptors for human health exposure assessment assigned to them.

Generic exposure scenario		Worker contributing scenario		
		Use descriptor	Contributing scenario	Scope derived from the ECPA OWB model
Professional use	1. Use as a co- formulant in plant protection products, spray applications by professionals	PROC 8a	Mixing and loading of plant protection products into delivery equipment	Loading of tractor-mounted/trailed boom sprayers, loading of tractor-mounted/trailed broadcast air-assisted sprayers, and loading of hand-held spray equipment.
		PROC 11	Delivery and dispersion of plant protection products	Tractor-mounted/trailed boom sprayers, tractor-mounted/trailed broadcast air- assisted sprayers, use of hand-held spray equipment for high-level targets (including greenhouses), worker re-entry (indirect exposure), and bystanders (indirect exposure).
	2. Use as a co-formulant in plant protection products, seed and granular applications by professionals	PROC 8a	Mixing and loading of plant protection products into seed treatment or delivery equipment	Loading of tractor-mounted broadcast spreaders, the loading of mechanical equipment with solid and liquid products for the treatment of seeds, and the loading of manual belly grinders and push spreaders.
		PROC 8b	Transfer of treated seeds from batch treater into bags	Transfer of chemicals from/to vessels/large containers at dedicated facilities.
		PROC 8a	Delivery and dispersal of agrochemical plant protection products or treated seeds	Manual spreading (by hand), mechanical spreading (belly grinders and push rotary spreaders), and from open-cab tractor- mounted broadcast spreaders.
Consumer use (‘amateur’)	3. Use as a co- formulant in plant protection products, spray applications by consumers	PC 27	Spray application of agrochemical plant protection products	Loading of hand-held spray equipment. Use of hand-held spray equipment for high-level targets.
	4. Use as a co-formulant in plant protection products, seed and granular applications by consumers	PC 27	Manual spreading of granular plant protection products or treated seeds	Manual spreading by hand/spoon/cup, push rotary spreader, or belly grinder, of granular plant protection products or treated seeds on residential lawns/turf, gardens (flowers, fruits, vegetables), and trees (fruits, nuts, shrubs, ornamentals).

### GES1: spray applications by professionals

At the time of the OWB model development, two exposure models for foliar spray application were considered, the German BBA model, and the UK POEM. The BBA model uses the area application rate (kg substance per hectare) as the only exposure determinant associated with a given application technique, mitigated by PPE selection. In contrast, POEM also requires the water dilution rate of the spray solution as an input parameter. To avoid assumptions on dilution and simplify the identification of a worst-case application rate for a given co-formulant, the BBA model was selected for spray applications. To support a single GES covering all aspects of spray applications, additional exposure models were introduced to extend the applicability domain of OWB beyond those activities covered by the BBA model, to greenhouses, worker re‐entry, and bystanders.

The BBA model is based on exposure studies generated in the 1980s. Technical progress and its exposure-reducing aspects during the last 30 years, such as closed cabins and drift-reducing technologies, have not been incorporated into the model. Long-term downward trends in inhalation exposures over time have been reported for other sectors (Creely *et al*., 2007). Because of this inherent conservatism, the geometric mean values have been accepted by European regulators for the assessment of active substances under PPP legislation. Following on from this established approach, the BBA model with geometric means was also considered to be appropriate for modelling of reasonable worst-case exposure of workers to co-formulants used in spray applications under REACH.

### GES1 contributing scenario: mixing and loading

The BBA model explicitly provides exposure predictions for mixing and loading of PPP, and considers the material transfer from a container to a receiving vessel. Exposure predictions are available for different application methods (tractor-mounted ground-boom spraying onto low crops, air-blast spraying, and hand-held spraying) and formulation types (liquid, granules, and powder).

Table 2 shows typical OWB output for mixing and loading (associated with spraying), which includes the calculated geometric mean exposure predictions for all permutations of the exposure determinants in the underlying BBA model. The worst-case tasks (considering hand-held separately) with the highest combined route exposure (RCR) are highlighted in bold, and are the values reported for the contributing scenario. Supplementary Equations S1 and S2, and Supplementary Tables S1 and S2, in the Supplementary Material (available at *Annals of Work Exposures and Health* online) provide details on the exposure calculations.

**Table 2.** Typical exposure estimate output for the generic exposure scenario 1, PROC8a, mixing and loading worker contributing scenario.

PROC 8a: Mixing and loading of plant protection products into delivery equipment^a^							
Type of equipment and conditions	Model	Formulation type	Personal protective equipment	Respiratory protective equipment	Dermal exposure (mg/kg bw/day)	Inhalation exposure (mg/m^3^)	Total risk characterization ratio
**Mixing and loading tractor-mounted/ trailed boom sprayer**	**BBA**	Liquid	**No PPE**	**No RPE**	0.793	0.0014	0.264
		**Powder (WP**)^**b**^			**1.982**	**0.1618**	**0.675**
		Granule (WG)^c^			0.661	0.0185	0.222
Mixing and loading tractor-mounted/ trailed broadcast air- assisted sprayer	BBA	Liquid	No PPE	No RPE	0.317	0.0006	0.106
		Powder (WP)			0.793	0.0647	0.270
		Granule (WG)			0.264	0.0074	0.089
**Mixing and loading hand-held sprayer, outdoors or indoors**	**BBA**	**Liquid**	**No PPE**	**No RPE**	**2.249**	**0.0038**	**0.750**
		Powder (WP)			0.549	0.0614	0.188
		Granule (WG)			0.230	0.0015	0.077

^a^Values calculated by ECPA OWB v3.3 using the parameters in the footnote to Table 4. Exposures and determinants leading to the tasks with highest combined route exposure are highlighted in bold, and are the values selected for risk assessment. ^b^WP = wettable powder. ^c^WG = water dispersible granules. PPE refers to input options of gloves and/or coveralls; RPE refers to input options with protection factors of 10 or 20.

### GES1 contributing scenario: application by spraying

Exposure from spray application of PPP is independent of the initial formulation types since dilution in water occurs before spraying. The BBA model covers tractor-mounted ground-boom spraying onto low crops, tractor-mounted air-blast spraying, and hand-held spraying of high targets (e.g. in orchards). Tractor-mounted and hand-held application methods were grouped and reported separately, to allow further refinement of the exposure scenario if required (i.e. easy separation into two contributing scenarios).

Table 3 shows typical OWB output for the spray application contributing scenario, which includes the models and calculated geometric mean exposure predictions for all permutations of the exposure determinants. The task with the highest combined route exposure (i.e. RCR) is highlighted in bold, and is the value reported for the contributing scenario. OWB calculations for Table 3 also use Supplementary Equations S1 and S2, and the values given in Supplementary Table S1 and S2 (available at *Annals of Work Exposures and Health* online).

**Table 3.** Typical exposure estimate output for the Generic Exposure Scenario 1, PROC11 spray application contributing scenario.

PROC 11: Delivery and dispersion of plant protection products^a^							
Type of equipment and conditions	Model	Formulation type	Personal protective equipment	Respiratory protective equipment	Dermal exposure (mg/kg bw/day)	Inhalation exposure (mg/m^3^)	Total risk characterization ratio
Tractor-mounted/ trailed boom spraying	BBA	Liquid	No PPE	No RPE	0.674	0.0023	0.225
**Tractor-mounted/ trailed broadcast air- assisted spraying**			**No PPE** ^**b**^	**No RPE**	**1.519**	**0.0166**	**0.508**
Hand-held spraying, high-level target, outdoors			No PPE	No RPE	0.443	0.0230	0.150
Hand-held spraying, high-level target, indoors (greenhouses)	BBA	Liquid	No PPE	No RPE	0.443	0.0230	0.150
Worker re-entry (indirect exposure)	Hoernicke *et al*. (1998)	Liquid	-	-	0.8778	-	0.293
Indirect exposure of bystanders	Martin *et al*. (2008)	Liquid	-	-	0.1512	0.0102	0.105^c^

^a^Values calculated by ECPA OWB v3.3 using the parameters in the footnote to Table 4. ^b^Exposure and determinants leading to the task with highest combined route exposure is highlighted in bold and are the values selected for risk assessment. PPE refers to input options of gloves and/or coveralls; RPE refers to input options with protection factors of 10 or 20. ^c^RCR is calculated using DNEL_general_.

### Extending the applicability domain to greenhouses

Volatile co-formulants can be used in greenhouses, and vapours could significantly contribute to inhalation exposure in addition to spray mist. Exposure to volatiles in enclosed spaces is outside the applicability domain of the standard BBA model, which is based on the data collected outdoors. To expand the applicability domain of OWB to this hand-held spraying scenario, the constant rate release model (CRRM) was used as implemented in ConsExpo (Delmaar *et al*., 2005). The CRRM requires only a limited number of input parameters: it assumes that a defined quantity of volatile substance (defined as vapour pressure >0.1 Pa) is instantaneously vaporized on release from the spray nozzle and distributed to the airspace (defined by greenhouse height) for a given duration. Removal of the substance from the greenhouse air by ventilation is taken into account. For non-volatile substances, only the BBA model is used for the greenhouse exposure estimate (as in the example in Table 3), however, the CRRM component is added for vapour pressures >0.1 Pa. The time-weighted average exposure is given by equation 1:

$$T\text{ hour TWA}=\frac{1}{T}\cdot \left(\frac{AR}{q\cdot h\cdot tr}\cdot t+\frac{AR}{q^{2}\cdot h\cdot tr}\cdot e^{-qt}-\frac{AR}{q^{2}\cdot h\cdot tr}\right)\cdot 100$$ (1)

where q = number of air changes per unit time (h^−1^), V = volume of air in the greenhouse (m^3^), tr = duration of substance release to air (h), t = total worker exposure time (h), AR = application rate of the substance (kg.ha^−1^), h = height of the greenhouse (m), T-hour TWA = time-weighted average (mg.m^−3^), and T = period over which the exposure is averaged (h). Further details on the derivation of equation 1 using the CRRM are given in the Supplementary Material (Supplementary Equations S3–S7 and Supplementary Table S3, available at *Annals of Work Exposures and Health* online).

### Extending the applicability domain to worker re-entry and bystanders

Dobe *et al*. (2017) describe the practical considerations made in creating additional specific contributing scenarios versus broadening the applicability domain of an exposure model, by taking into account additional tasks. Although neither worker re-entry nor exposure of bystanders (general population) is a formal use of a substance, they are a potential consequence of spraying, and thus are linked to this professional contributing scenario. OWB applicability domain was extended to cover worker re-entry by utilizing an approach previously used in PPP assessments (Krebs *et al*., 2000). In this model, the exposure is dependent on the dislodgeable foliar residues (DFR), transfer coefficient from leaves to worker (TC), work rate, and application rate of the substance. For co-formulants which are volatile (≥0.1 Pa), the DFR is assumed to be negligible 24 h after application due to complete evaporation of the substance. The DFR value used in OWB was based on an average literature value (Brouwer *et al.*, 2000), and assuming two applications. The transfer coefficient used was the second highest value from the EUROPOEM dataset (van Hemmen *et al*., 2002). Further explanation of the model derivation is given in the Supplementary Material (Supplementary Equation S8 and Supplementary Table S4, available at *Annals of Work Exposures and Health* online).

The OWB applicability domain was extended to cover bystanders using an approach previously used for the assessment of PPP (Martin *et al*., 2008). In this model, the dermal exposure is dependent on the application rate, spray drift rate, exposed body area, and body weight. The value used for spray drift was a reasonable worst-case from Rautmann *et al*. (2001). The inhalation exposure is dependent on the application rate, unit exposure per kilogram of the substance handled (Lundehn *et al*., 1992), respiratory volume, and duration considered. Additional inhalation exposure from vapour is considered for volatile substances. It should be noted that the general population DNEL must be used for this RCR calculation, although this is formally a professional worker exposure scenario. Exposure of bystanders is typically less than for workers, nevertheless, if such exposure were to limit the maximum use rate of a co-formulant (the DNEL_general population_ used in the RCR calculation is more conservative), this restriction must be reflected in the professional rather than a separate contributing scenario. Further details are given in the Supplementary Material (Supplementary Equations S9 and S10 and Supplementary Tables S5 and S6, available at *Annals of Work Exposures and Health* online).

### Combined exposure for spray applications

Mixing and loading, and spraying of PPP are correlated contributing scenarios, because they are usually carried out in sequence by the same workers. The application technique resulting in the highest combined exposure from the underlying correlated tasks is identified as worst case. The use rate maximization routine implemented in OWB uses this largest combined task exposure to maximize the application rate, rather than the sum of the contributing scenarios, as this could lead to over-prediction of exposure. For example, without PPE the broadcast air-assisted spraying task has the highest predicted exposure for the PROC 11 contributing scenario (Table 4). For PROC 8a, this lies with the mixing and loading for trailed boom spraying. Both are reported as the worst-case exposures for the respective contributing scenarios. However, when combined exposure is considered, the trailed boom-spraying task has the highest overall predicted exposure. For this reason, the combined exposure predictions differ from a simple addition of the PROC 8a and PROC 11 contributing scenarios, as would be the case if the underlying BBA model reported application method independent mixing and loading exposures.

**Table 4.** Typical combined task exposure estimates for generic exposure scenario 1 (spray application) and used for maximum use rate calculations.

Contributing Scenarios	Max use rate^a^		Dermal exposure (mg/kg bw/day)	Inhalation exposure (mg/m^3^)	Personal/respiratory protective equipment		Risk characterization ratio		
	(kg/ha)	(kg/d)					Dermal	Inhalation	Total
**Tractor-mounted boom spraying**									
PROC 8a: Mixing & loading WP formulation	**1.16**	**23.12**	1.982	0.1618	No PPE	No RPE	0.661	0.015	0.675
PROC 11: Tractor-mounted boom spraying			0.674	0.0023	No PPE	No RPE	0.225	0.000	0.225
**PROC 8a+11**			**2.655**	**0.164**			**0.885**	**0.015**	**0.900** ^**b**^
**Tractor-mounted air-blast spraying**									
PROC 8a: Mixing and loading WP formulation	1.16	9.25	0.793	0.0647	No PPE	No RPE	0.264	0.006	0.270
PROC 11: Tractor-mounted air-blast spraying			1.519	0.0166	No PPE	No RPE	0.506	0.002	0.508
**PROC 8a+11**			2.312	0.081			0.771	0.007	0.778
**Hand-held spraying**									
PROC 8a: Mixing & loading liquid formulation into knapsack sprayer	**0.77**	**0.77**	2.249	0.004	No PPE	No RPE	0.750	0.000	0.750
PROC 11: Hand-held spraying, indoors (greenhouse)			0.443	0.023	No PPE	No RPE	0.148	0.002	0.150
**PROC 8a+11**			**2.693**	**0.027**			**0.898**	**0.002**	**0.900** ^**b**^

^a^Values calculated by ECPA OWB v3.3 for maximized use rate of a co-formulant for a target RCR = 0.9; 1.16 kg/ha (tractor) and 0.768 kg/ha (hand-held). Input parameters: physical state = solid, vapour pressure = 0.001 Pa, DNELworker,long-term,inhalation = 11 mg/m^3^, DNELworker,long-term,dermal = 3 mg/kg bw, DNELgeneral,long-term,inhalation = 2.6 mg/m^3^, DNELgeneral,long-term,dermal = 1.5 mg/kg bw, no PPE.

^b^Values highlighted in bold are the maximum use rate, exposures, and resulting RCRs used in the use rate maximization calculation.

### GES2: seed and granular application by professionals

Predictions for this exposure scenario cover the professional use of a co-formulant in PPP, applied as granules, or treated seeds, in indoor and outdoor environments. The Pesticide Handler Exposure Database (PHED) as presented in the Occupational Pesticide Handler Unit Exposure Surrogate Reference Table (US EPA, 2013), was selected to assess the handling of granular materials in the absence of a corresponding European model. Proprietary models considering worker exposure to active ingredients arising from tasks associated with seed treatment activities are available; however, because these are not in the public domain, surrogate values describing similar activities were used.

### GES2 contributing scenario: mixing and loading

This contributing scenario includes loading of the concentrated PPP into a process tank for seed treatment and the loading of treated seeds or granular PPP into delivery equipment. Typical exposure and model output for this contributing scenario is shown in Supplementary Table S7 in the Supplementary Material (available at *Annals of Work Exposures and Health* online).

Supplementary Equations S1 and S2 are used to calculate exposure via dermal and inhalation routes, using default values given in Supplementary Tables S8 and S9 in the Supplementary Material (available at *Annals of Work Exposures and Health* online). The exposure resulting from loading of hand-held equipment (push-type rotary spreaders and belly grinders) cannot be estimated separately by the PHED for hand-held equipment. Use of working clothes (long-sleeve shirt, long pants, shoes plus socks) is assumed in the PHED exposure predictions.

Seed treatment is normally conducted as an industrial process at designated facilities. To consider on-farm/small-scale treatment, the transfer of PPP into a seed treatment process tank was assessed using an analogous task from the BBA model: loading of liquid or WP formulations into a tractor-mounted tank.

### GES2 contributing scenario: transfer of treated seeds

After treatment seeds are transferred into bags for storage and transport at dedicated industrial facilities. Exposure from bagging of the treated seeds was assessed using ECETOC TRA v3.1. The input parameters selected are given in Supplementary Table S10, and typical OWB output is given in Supplementary Table S11 in the Supplementary Material (available at *Annals of Work Exposures and Health* online).

### GES2 contributing scenario: delivery and dispersal of granules and seeds

The PHED was used to assess exposure resulting from granular PPP or treated seeds spread by hand, or using hand-held or tractor-mounted equipment (see Supplementary Tables S12 and S13 in the Supplementary Material, available at *Annals of Work Exposures and Health* online). This model considers hand-held belly grinders, push-type rotary spreaders, and tractor-mounted broadcast spreaders. Use of working clothes (long-sleeve shirt, long pants, shoes plus socks) is assumed in the exposure predictions. Granules and treated seeds are generally not expected to contain liquids or volatile substances, removing the need for separate indoor scenarios.

### Combined exposure for seed and granular application

Mixing and loading of seed treatment PPP, and bagging of treated seeds, are correlated tasks. Similarly, mixing and loading as well as dispersion of seeds and granules are correlated. Both are considered in the application rate maximization routine (see Supplementary Table S14, available at *Annals of Work Exposures and Health* online).

### GES3 and GES4 for consumer uses

Applications of PPP by consumers are also covered by OWB. The models for spray and solid applications by workers are adapted to the consumer scenarios by assuming no PPE is available; treated areas in private homes and gardens do not exceed 200 m^2^/day; body weights are 60 kg instead of 70 kg (ECHA, 2012); and bearing in mind the conservatism in the BBA geometric means the 75th percentile values are used to reflect higher exposure potential for untrained spray users. For the assessment of granules and treated seeds, the US EPA Standard Operating Procedures for Residential Exposure Assessments (SOPREA) was used (US EPA, 2012). See Supplementary Tables S15, S16, and S17 in the Supplementary Material, available at *Annals of Work Exposures and Health* online.

### Benchmarking

The relevant models upon which OWB is based were benchmarked for professional uses to demonstrate equivalence: German BBA and PHED, as well as the new EFSA AOEM for comparison. Benchmarking was also carried out against the tier 1 ECETOC TRA (version 3.1, considered to be 75th percentile).

A powdered formulation containing a hypothetical solid substance with a vapour pressure of 0.001 Pa, and a liquid formulation containing a hypothetical liquid substance with vapour pressure of 0.001 Pa were used as a basis for the benchmarking calculations.

The AOEM inputs were chosen to match as closely as possible the worst-case task identified by the OWB tool. For benchmarking calculations the co-formulant concentration in concentrated PPP and diluted spray solutions is assumed to be 100% and 1%, respectively. To permit reproduction of the benchmarking calculations, full details on input parameters are given in Supplementary Tables S18–S25 in the Supplementary Material (available at *Annals of Work Exposures and Health* online).

OWB is primarily designed to generate a maximum application rate for a specific co-formulant. However, for benchmarking, a standard application rate of 1 kg/ha was selected to permit a like-with-like comparison across models, although in particular for hand-held spraying this is normally constrained to a rate less than for tractor-mounted applications. No PPE was assumed to permit easier comparison of predicted exposure without the complication of varying protection factor (PF) assignments. Whereas application rates per hectare are well-defined for PPP, the duration of the mixing, loading, and application tasks on the day of use is often unknown. This introduces significant uncertainty into the exposure estimate comparison between the PPP and REACH models, where duration for the latter is a key exposure determinant. Based on expert judgement, cumulative 60 min per work day was assumed for mixing and loading activities, 360 min for hand-held application activities, and 480 min for mechanically assisted application activities. Calculations were not performed for the liquid substance for the GES2 contributing scenarios, as this is usually not relevant for the solid state (granules and treated seeds).

## Results

Table 4 illustrates typical OWB output for the spray application GES1, showing the results of the combined task exposure calculations from Tables 2 and 3. The two highest exposure activities are highlighted in bold, and used for combined exposure risk characterization, and to determine the maximum application rate of the substance to be communicated to the downstream user as an OC in the extended safety data sheet.

No PPE has been selected as input in the Table 4 example, thus giving the worst-case (smallest) maximum application rate for the co-formulant. For context, 2 kg/ha is a typical PPP spray application rate, so the hypothetical co-formulant in Table 4 could reasonably be used in concentrations to ca. 50% for tractor-mounted applications. Use of gloves at least for mixing and loading tasks could be expected for all PPP, and thus if specified as a RMM, would lead to an increased maximum use rate for the co-formulant (e.g. useful for solvents), with limited likelihood of conflicting with the labelling and RMM of existing PPP authorizations. Exposure of workers entering a treated field (re-entry) or bystanders is not shown separately in Table 4, however, the respective RCRs are taken into consideration when the maximum use rate of the co-formulant is determined.

Seed treatment and the dispersal of granules or treated seeds are not performed by the same individuals, and consequently, the RCRs from these contributing scenarios are not combined. Tractor-mounted broadcast spreading of granules has a separate set of parameters for the loading activity, and for mechanical spreading exposure from loading tasks is implicit in the model used. Supplementary Table S14 (available at *Annals of Work Exposures and Health* online) shows typical results for seed treatment, bagging, and dispersion of granules.

### Benchmarking

**Figure 1.**
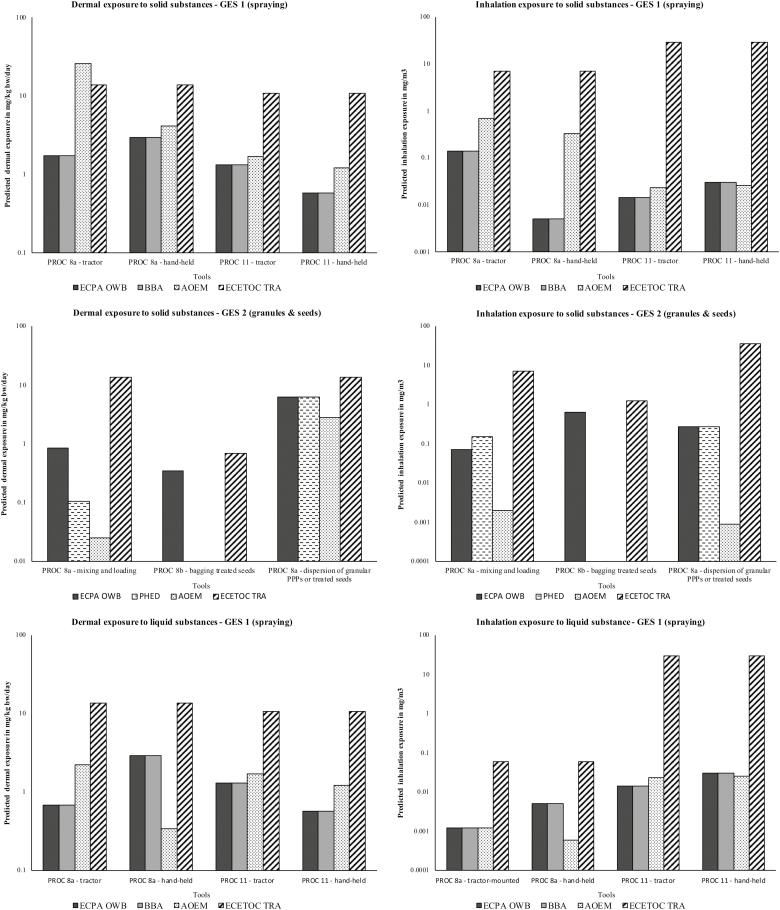
Results of the exposure model benchmarking calculations for generic exposure scenario 1 and 2 contributing scenarios. Values are for: powder VP = 0.001 Pa/liquid VP = 0.001 Pa at 1 kg/ha use rate. ECPA OWB values represent the worst-case exposures calculated from the underlying PPP models, and are compared against predictions for the same scenario using the AOEM and ECETOC TRA models.

The results of benchmarking OWB against ECETOC TRA, and the PPP models BBA, PHED and AOEM, for the professional exposure scenarios GES1 and GES2 are shown in Fig. 1 (see also Supplementary Table S26, available at *Annals of Work Exposures and Health* online). OWB gave equivalent exposure predictions to the BBA and PHED models, with the exception of GES2 PROC8a (mixing and loading), which uses the BBA rather than PHED for worst-case exposure estimation.

For spray application (PROC11), the AOEM inhalation prediction was similar to OWB, however, particularly for scenarios involving mixing and loading of powders (PROC8) some predictions were significantly higher, and in one case exceeded that of ECETOC TRA (GES1, dermal exposure, tractor-mounted). Conversely, for other mixing and loading, and dispersal scenarios, the worst-case AOEM predictions were significantly smaller than OWB, and no overall pattern in conservatism could be distinguished. Apart from the above exception, ECETOC TRA predicted higher exposures than the PPP-specific models (and excessively so for PROC11), which would be the desired conservative outcome from a tier 1 model used for screening purposes.

## Discussion

OWB is largely based on the existing exposure models that have been used for the authorizations of PPP in Europe. Because they are based on measured data involving mixing and loading, and application of PPP, they provide a potentially more realistic exposure estimate compared to predictive models based on industrial worker exposure data. While the tasks are fundamentally the same (e.g. transfer, spraying, spreading), systematic differences in typical industrial and PPP handling and exposure could arise from various differences in e.g. packaging, risk perception, spray drift reduction technologies, etc. The models were simplified for use in the OWB screening level tool, typically by making reasonable worst-case assumptions to reduce information requirements.

The heavy tailoring of OWB to the REACH registration requirements facilitates the use of the PPP-specific exposure models by risk assessors working outside the PPP industry (e.g. co-formulant manufacturers), while at the same time enabling downstream users (PPP manufacturers) to readily generate refined risk assessments for use in downstream user CSRs. OWB includes a feature that automatically produces pre-populated templates for the relevant sections of the CSR, with the intent to increase standardization, efficiency, and transparency of the exposure assessment for both end user and regulators.

While the worker exposure models typically used for REACH may be relevant for tasks involving the transfer and mixing of PPP, those tasks involving the dispersal of PPP, e.g. spraying, or spreading of granules, are more likely to be outside the intended applicability domains of those models. On the other hand, the standard higher-tier exposure models developed specifically for PPP have many exposure determinants for which a registrant will typically not have detailed information, potentially leading to significant variability between risk assessments, and potentially conflicting or spurious RMM obligations between suppliers for the same co-formulant. It should be remembered that the typical inputs required for the registrant’s exposure assessment of a co-formulant with these models are the use (crop, maximum application rate, and application method) and formulation (type, dermal absorption, and concentration) details for all the use-formulation pairs containing the substance. Furthermore, these details are spread across all companies holding relevant product authorizations, and for the same formulations, these inputs can vary according to the specifics of the authorization in each of the EEA countries. OWB addresses this information deficit by making available an exposure model that simultaneously assesses all relevant scenarios and provides the worst-case exposure for use in a standardized risk assessment approach. This is likely to be especially helpful for registrants unfamiliar with PPP assessments, as well as for downstream users who may as a result receive a standardized and meaningful exposure scenario and maximum use rate which can be compared with their existing PPP use patterns.

OWB should be used with care for volatile co-formulants e.g. solvents. The underlying BBA model is based on the data collected for 16 active substances (Lundehn, 1992), which have low vapour pressures (highest is dinoseb acetate with 0.08 Pa), and thus reflects exposure to aerosols. Predicted dermal exposures for volatile co-formulants can be expected to be worst-case, because volatilization from spray mist before deposition (Delmaar and Bremmer, 2009), as well as subsequent volatilization from the skin, is not accounted for. Inhalation of vapours is considered in addition to spray mist in the greenhouse and bystander exposure predictions for vapour pressures >0.1 Pa. As a result, if the maximum application rate is reported as an OC and limited to hand-held spraying exposure (Table 4), then more volatile substances can be considered. However, inhalation of vapour is not considered for tractor-mounted spray methods for vapour pressures >0.1 Pa. Recently reported data suggests that the cross-over from primarily aerosol to vapour exposure occurs with vapour pressures around 10–100 Pa (FRAUNHOFER, 2018), higher than previously suggested 0.01–0.1 Pa (Bremmer *et al*., 2006). This preliminary data suggests that models based on aerosol exposure may be useful for substances with higher vapour pressures than 0.1 Pa, but further work is required in this area.

The benchmarking results in Fig. 1 show that OWB and BBA geometric mean exposure predictions for PROC11 are similar to the more recently introduced AOEM 75th percentile predictions. This supports the position previously taken by PPP regulators that due to technical progress in spray application methods, use of BBA geometric means was acceptable. Greater variation is seen in the transfer (PROC8) predictions, with the AOEM predicting both higher and lower exposures in comparison.

For the inputs selected to model the benchmarking scenarios, the tier 1 model ECETOC TRA clearly delivered more conservative inhalation and dermal exposure estimates than the models developed specifically for regulatory PPP assessments: OWB (BBA and PHED) or AOEM models. However, while ECETOC TRA may overall deliver worker exposure estimates which are conservative, its use for PPP worker exposure assessments should nevertheless be discouraged, particularly where prediction of inhalation exposure (PROC 11) leads to stipulation of restrictive RMM such as RPE, or on maximum concentration or duration of use. Such RMM potentially conflict unnecessarily with the existing PPP authorizations (labelling and required PPE), thus triggering the need for a downstream user CSR and use of a more suitable model. Finally, it should also be remembered that the standard REACH models do not consider worker re-entry, or potential bystander exposure.

The approach taken in constructing the OWB is extensible, permitting incorporation of additional or replacement exposure models to expand or update the applicability domain as required. The recently released AOEM model for the harmonized assessment of worker exposure to PPP has superseded some of the models on which OWB was based for PPP regulatory use in Europe. However, being a higher-tier model, due to the input information requirements, it is unlikely to be suitable for general co-formulant assessment in the REACH context, other than by PPP manufacturers in a downstream user CSR situation. Even in this context, many of the simplifications made in OWB would have to be made manually to use the AOEM, requiring further expertise and increasing inter-user assessment variability. Given that the underlying algorithms and data for the AOEM model are publically available, it is planned to update the relevant models used in OWB to maintain alignment with PPP regulatory assessments, using the same approaches described in this paper.

## Conclusions

OWB as used to date in REACH registrations generates exposure predictions for co-formulants in PPP equivalent to existing PPP models, when these are configured for reasonable worst-case predictions. The model has been designed for regulatory use, to closely integrate with the ECPA GES contributing scenarios, with broad and transparent applicability domain, and to provide worst-case screening level exposure predictions for each exposure scenario. OWB aims to facilitate the routine assessment of co-formulant uses in PPP under REACH, and thereby aid the continued availability of chemicals that are essential for effective crop protection products.

## Supplementary Material

Supplementary Materials
