# Supplementary material for: REACH Worker Exposure Model for Co-formulants Used in Plant Protection Products
Source: Ann Work Expo Health. 2018 Oct 31;63(1):54–67. doi: 10.1093/annweh/wxy088 (PMC6356032; doi:10.1093/annweh/wxy088)
Supplement: Supplementary Materials [file wxy088_suppl_supplementary_material.pdf]

**Online Supplementary Material**

# REACH Worker Exposure Model for Co-Formulants Used in Plant Protection Products


Volker Mostert [1], Sebastien Bonifay [2], Christopher Dobe [3], Ralph Fliege [4], Joachim Krass [5], Renate Vosswinkel [4], Matthias Wormuth [3]

[1] extera, Nelly-Sachs-Str. 37, 40764 Langenfeld, Germany
[2] DuPont de Nemours, Bedrijvenlaan 9, B2800 Mechelen, Belgium
[3] Syngenta Crop Protection AG, Schwarzwaldallee 215, 4058 Basel, Switzerland
[4] Bayer AG, Crop Science Division, Alfred-Nobel-Str. 50, 40789 Monheim, Germany
[5] BASF SE, Carl-Bosch-Str. 38, 67056 Ludwigshafen, Germany

**Corresponding Author:**
Dr Christopher Dobe
Syngenta Crop Protection AG, Schwarzwaldallee 215, 4058 Basel, Switzerland
Tel.: +41-613238557
Email: christopher.dobe@syngenta.com

## GES1: SPRAY APPLICATIONS BY PROFESSIONALS

The applicability domain of the OWB GES1 exposure predictions is derived from the underlying models. It covers the transfer of solid and liquid PPPs which occurs during loading of tractor-mounted tanks or knapsack sprayers; the spray application of PPPs using tractor-mounted boom sprayers, tractor-mounted air-blast sprayers, and hand-held spray equipment for high-level targets including in greenhouses; and the indirect exposure of workers on field re-entry and of bystanders. Further details on these models are given below.

The BBA model assumes 100% transmission to skin, however, for mixing and loading, gloves with a protection factor (PF) of 100 are an optional RMM (Lundehn et al., 1992). Equations S1 and S2 are implemented in the OWB:

$$E_{\text{dermal}} = \frac{AR \cdot A \cdot UE_{\text{dermal}}}{PF \cdot BW}$$ **Equation S1**

and

$$E_{\text{inh}} = \frac{AR \cdot A \cdot UE_{\text{inhal}}}{PF \cdot RV}$$ **Equation S2**

Where $E_{dermal}$ = external dermal dose (mg/kg bw/day), $E_{inh}$ = inhalation exposure (mg/m$^3$), AR = application rate (kg/ha), A = Area (ha), $UE_{route}$ = unit exposure for the relevant route and quantity handled (mg/kg substance handled), BW = body weight (kg), RV = respiratory volume (m$^3$/8 h), PF = protection factor.

**Contributing scenario: mixing and loading**

**Table S1. Default unit exposure ($UE_{route}$) estimates for workers adapted from Lundehn et al. (1992). Exposure values are geometric means.**

| Contributing Scenario | Unit exposure values (mg substance / kg substance used)[a] | | | | | | | | |
|---|---|---|---|---|---|---|---|---|---|
| | Hand-held sprayer | | | Ground boom sprayer | | | Air-blast sprayer | | |
| | Liquid | WG[b] | WP[c] | Liquid | WG[b] | WP[c] | Liquid | WG[b] | WP[c] |
| **Mixing and Loading [PROC 8a]** | | | | | | | | | |
| Dermal: Hand | 205 | 21 | 50 | 2.4 | 2.0 | 6 | 2.4 | 2.0 | 6 |
| Inhalation | 0.05 | 0.02 | 0.8 | 0.0006 | 0.008 | 0.07 | 0.0006 | 0.008 | 0.07 |
| **Spraying [PROC 11]** | | | | | | | | | |
| Dermal: Head | 4.8 | | | 0.06 | | | 1.2 | | |
| Dermal: Hand | 10.6 | | | 0.38 | | | 0.7 | | |
| Dermal: Rest of body | 25 | | | 1.6 | | | 9.6 | | |
| Inhalation | 0.3 | | | 0.001 | | | 0.018 | | |

[a] Exposure values are geometric means. [b] Water-dispersible granule. [b] Water-soluble powder.

**Table S2. Default parameters for use in Equations S1 and S2, for the contributing scenarios mixing and loading, and application by spraying.**

| Variable | Value | Comment |
|---|---|---|
| *AR* | 1 kg.ha$^{-1}$ (or maximized) | Default application rate. Can be specified or maximized to a specified RCR. Tractor and hand-held application methods are not constrained to the same AR. |
| *A* | 20 ha (boom sprayer)<br>8 ha (air blast)<br>1 ha (hand-held) | Boom spraying has a much higher daily work rate than air-blast spraying (orchards). |
| *UE* | See Table S1 | $UE_{dermal}$ uses the sum of head, hand, and rest of body for PROC 11. |
| *BW* | 70 kg | REACH default (ECHA 2012). |
| *RV* | 10 m$^{3}$·day$^{-1}$ | REACH default for professional light work (ECHA 2012). |
| *PF* | None<br>100 | Unless specifically specified, the BBA model assumes no protective clothes are worn, i.e. workers wear light clothes consisting of T-shirt, shorts and shoes. |

**Contributing scenario: application by spraying**

**Extending the model to greenhouses (indoor spraying)**

The constant rate release model (CRRM) assumes a known quantity of substance is released continuously over a defined period in an enclosed space. It is assumed that the substance is immediately volatilized on release from the spray nozzle. The concentration increases during the application, while simultaneously being removed by natural ventilation. The following equation for calculating the concentration in air at a given time was used (Delmaar et al., 2005, Equation 3a):

$$C_{\mathrm{air}} = \frac{A_0 \cdot Wf}{q \cdot V \cdot tr} \cdot \left(1 - e^{-qt}\right)$$ **Equation S3**

Equation S3 was integrated for use in the ECPA OWB model:

$$C_{air} = \frac{A_0 \cdot Wf}{q \cdot V \cdot tr} \cdot t + \frac{A_0 \cdot Wf}{q^2 \cdot V \cdot tr} \cdot e^{-qt} - \frac{A_0 \cdot Wf}{q^2 \cdot V \cdot tr}$$ **Equation S4**

where:

| | |
|---|---|
| $C_{\mathrm{air}}$ = concentration of the substance in the greenhouse air | [kg.m$^{-2}$] |
| $A_0$ = mass of the substance applied | [kg] |
| *Wf* = weight fraction of the substance in the formulation applied | [fraction] |
| *q* = number of air changes per unit time | [h$^{-1}$] |
| *V* = volume of air in the greenhouse | [m$^3$] |
| *tr* = duration of substance release to air | [h] |
| *t* = total exposure time | [h] |

For the greenhouse scenario where exposure and substance release period are correlated, and correspond only to spraying activity within the greenhouse, $C_{air}$ = 0 mg m$^{-3}$ at t = 0 h, and t = $t_r$.

Equation S4 can be further simplified to give Equation S5 using the following relations: *Mass = Rate x Area* and *Volume = Area x Height*, assuming the full greenhouse area is treated (if not Equation S5 would overestimate exposure). Because only the substance application rate is considered, the weight fraction term is redundant i.e. *Wf* = 1.

$$C_{air} = \frac{AR}{q \cdot h \cdot tr} \cdot t + \frac{AR}{q^2 \cdot h \cdot tr} \cdot e^{-qt} - \frac{AR}{q^2 \cdot h \cdot tr}$$ **Equation S5**

where:

| | |
|---|---|
| *AR* = application rate of the substance | [kg.ha$^{-1}$] |
| *h* = height of the greenhouse | [m] |

The time weighted average is given by:

$$T\text{ hour TWA} = \frac{1}{T} \cdot \left( \frac{AR}{q \cdot h \cdot tr} \cdot t + \frac{AR}{q^2 \cdot h \cdot tr} \cdot e^{-qt} - \frac{AR}{q^2 \cdot h \cdot tr} \right) \cdot 100$$ **Equation S6**

where:

*T* hour TWA = time weighted average, e.g. 8-h TWA [$mg.m^{-3}$]
*T* = period over which the exposure is averaged [h]
100 = conversion factor $kg.ha^{-1}$ to $mg.m^{-2}$

From Equation S6 it is apparent that the worker exposure to a volatile substance will depend only on the application rate, ventilation rate, height of the greenhouse, and exposure duration. The exposure is independent of vapour pressure because it is assumed for substances with a vapour pressure >0.1 Pa that complete volatilisation is immediate.

**Table S3. Parameters used in the CRRM to estimate exposure to volatile co-formulants in a greenhouse.**

| Variable | Value | Comment |
|---|---|---|
| *tr = t* | 6 h | Duration is the standard assumption used in crop protection exposure assessments, and represents a worst-case of 6h spraying within a single contiguous greenhouse. Exposure is assumed to stop after leaving the greenhouse. |
| *T* | 8 h | A standard 8 hour working day is used for DNEL comparison. |
| *h* | 3 m | Height is the standard value assumed for crop protection greenhouse assessments. The concentration of the volatile substance increases with a lower greenhouse height, due to the smaller volume of air available for dilution of the unit application rate. |
| *AR* | 1 $kg.ha^{-1}$ (or maximized) | Default application rate (or maximized to achieve a specified RCR). |
| *q* | 1 $h^{-1}$ | Natural ventilation only has been assumed. Mechanically ventilated greenhouses would have a higher exchange rate. |

Using Equation S6 and the default parameters in Table S3 corresponding to a 1 ha greenhouse (V = 30 000$m^3$), the estimated exposure is given by the simple relation:

$TWA_{8h} = AR \cdot 3.47$ $mg/m^3$ **Equation S7**

where *AR* is the application rate of the co-formulant [kg/ha]. The total inhalation exposure for greenhouses where vapour pressure is >0.1 Pa is then calculated as the sum of the BBA and CRRM.

**Extending the model to worker re-entry**

Exposure of workers entering treated areas is predicted using an exposure model proposed by Hoernicke et al. (1998) and Krebs et al. (2000), whereby workers are potentially exposed to dislodgeable foliar residues (DFR), predominantly by dermal contact with the foliage. For volatile substances the DFR was assumed to be zero 24 hours after application. Considering the timescale, a vapour pressure cut-off of ≥0.1 Pa was used. Foliar residues depend on the application rate, residues from previous applications, and the Leaf Area Index (LAI) - the leaf area per unit ground surface area. The transfer of these residues from foliage to the clothes or skin of workers depends mainly on the intensity of the contact with the foliage, and is reflected in the Transfer Coefficient (TC). Activities with similar use patterns can be grouped to give a generic TC.

The unit DFR was estimated as a fixed value on the basis of the following assumptions: 1 kg substance/ha = 10 µg/cm², and two sided leaves reduces this to 5 µg/cm², and a LAI of ca. 3 - 5 further reduces this to 1 - 1.66 µg $cm^{-2}.kg^{-1}.ha^{-1}$. A generic DFR was thus estimated at 1 µg $cm^{-2}.kg^{-1}.ha^{-1}$ for row crops which do not cover the total sprayed area so that part of the spray is also deposited on the ground.

This value was also reported as a mean value by Brouwer et al. (2000). Re-entry activities (e.g., pruning) are most common for ornamentals that are treated using hand-held equipment, thus the hand-held application was used for the calculation of the DFR. As a simple approach to account for multiple applications, the field is assumed to receive two treatments from products containing the same co-formulants, without dissipation, thus doubling the hand-held application rate.

In accordance with the approach used by EUROPOEM II (van Hemmen et al., 2002), a TC of 5000 $cm^2$/h was chosen as a reasonable worst-case for a worker re-entering an area with treated ornamentals, wearing light clothes without gloves and having intensive skin contact with treated foliage. Skin contact is mainly with the palms of the hands, i.e. about 480 $cm^2$, and is used to estimate the local dose on skin.

The estimated dermal dose (*D*) is calculated using Equation S8:

$$D = DFR \cdot TC \cdot WR \cdot AR \qquad \textbf{Equation S8}$$

Where:

| | | |
|---|---|---|
| *DFR* = Dislodgeable foliar residues | [$\mu g\ cm^{-2}.kg^{-1}.ha^{-1}$] |
| *TC* = Transfer coefficient | [$cm^2.person^{-1}.h^{-1}$] |
| *WR* = Work rate | [$h.day^{-1}$] |
| *AR* = Application rate | [$kg.ha^{-1}$] |

The evaluation of exposure for a re-entry situation directly after application (spray deposit has dried with no dissipation) and using reasonable worst case values for each parameter, is considered to result in a very conservative approach.

The default values and assumptions used for the calculation of worker exposure occurring during re-entry are given in Table S4.

**Table S4. Default values for assessing dermal exposure to foliar residues.**

| Variable | Value | Comment |
|---|---|---|
| *DFR* | 1 $\mu g.cm^{-2}.kg^{-1}.ha^{-1}$ | As derived above. |
| *TC* | 5000 $cm^2.person^{-1}.h^{-1}$ | van Hemmen et al. (2002 ) |
| *WR* | 1 $ha.day^{-1}$ | Standard assumption in crop protection exposure assessments for maximum working area for a manual task. |
| *AR* | 1 $kg.ha^{-1}$ (or maximized) | Default application rate (or maximized to a specified RCR). |

**Extending the model to bystanders**

Bystander exposure involves potential exposure of the general public to drifting spray and vapour containing the substance in question.

The potential dermal exposure of bystanders is calculated using Equation S9:

$$E_{dermal} = \frac{AR \cdot Drift \cdot EA \cdot 100}{BW}$$ **Equation S9**

Where:

| | |
|---|---|
| $E_{dermal}$ = Dermal exposure | [mg/kg bw] |
| *AR* = Application rate | [kg/ha] |
| *Drift* = Drift rate | [%] |
| *EA* = Exposed body area | [m²/person] |
| *BW* = Body weight | [kg] |
| 100 = conversion factor from kg/ha to mg/m² | |

The potential inhalation exposure of bystanders is calculated using Equation S10:

$$E_{inh} = \frac{AR \cdot A \cdot UE \cdot D_{exp}}{RV \cdot D}$$ **Equation S10**

Where:

| | |
|---|---|
| $E_{inh}$ = Inhalation Exposure | [mg/m³] |
| *AR* = Application rate | [kg/ha] |
| *A* = Application area | [ha] |
| *UE* = Unit exposure per kg of substance handled | [mg/kg] |
| $D_{exp}$ = Duration of exposure to drift | [min] |
| *RV* = Respiratory volume | [m³] |
| *D* = Duration of spray full spray period | [min] |

The default values and assumptions used for the calculation of dermal and inhalation exposure are given in Table S5.

**Table S5. Default values for assessing of bystander exposure to spray drift.**

| Variable | Value | Comment |
|---|---|---|
| *AR* | 1 kg/ha | Default application rate (or maximized to a specified RCR). |
| *Drift* | 11.81% | The 90th percentile for spraying of early fruit crops (orchards) according to Rautmann et al. (2001). A distance of 10 m from the spray equipment was assumed. |
| *EA* | 1 $m^2$ | Adult wearing t-shirt and shorts. |
| *BW* | 60 kg | Default value. |
| *A* | 1 ha/day | Default spray area for the highest drift considered (hand-held, high target spraying). |
| *UE* | 0.3 mg/kg | Worst-case operator inhalation exposure taken for hand-held (knapsack) spraying of high crops (Lundehn et al., 1992) |
| $D_{exp}$ | 5 min/day | Assumed time bystanders spend in the vicinity of a drift event. |
| *RV* | 20 $m^3$ | Default respiratory volume of an adult member of the general public for 24 h period considered for TWA. |
| *D* | 360 min/day | Duration of the full spray period |

Following previous practice for the assessment of bystander exposure to active substances in PPP, vapour drift exposures (Table S6) are added to the spray drift exposure depending on the vapour pressure of the substance.

**Table S6. Default values for exposure to vapour drift** (Martin et al., 2008)

| Volatility class | Exposure to vapour drift [mg/m$^3$] |
|---|---|
| Non-volatile (VP < 1 × $10^{-5}$ Pa) | negligible |
| Semi-volatile (VP ≥ 1 × $10^{-5}$ Pa and < 5 × $10^{-3}$ Pa) | 0.001 |
| Volatile (VP > 5 × $10^{-3}$ Pa) | 0.015 |

## GES2: SEED AND GRANULAR APPLICATION BY PROFESSIONALS

The OWB applicability domain for GES2 resulting from the underlying models covers the transfer of treated seeds and granular PPPs, the loading of equipment for seed treatment with solid or liquid PPPs, loading of manual belly grinders and “push-type” rotary spreaders, transfer of seeds from batch treaters into bags, and dispersal of treated seeds and granular PPPs by hand, mechanical spreaders (belly grinders, push-type spreaders), or tractor-mounted broadcast spreaders. Further details on these models are given below.

Example calculations by ECPA OWB v3.3 are provided in Tables S7, S11, S13 and S14. The same input parameters given in the footnote to Table 4 have been used for consistency.

### Contributing scenario: Mixing and loading

**Table S7. Typical exposure estimate output for the GES2 PROC8a mixing and loading worker contributing scenario.**

| Type of equipment and conditions | Model | Formulation type | PPE | RPE | Dermal exposure [mg/kg bw/day] | Inhalation Exposure $[mg/m^3]$ | Total RCR |
|---|---|---|---|---|---|---|---|
| Mixing and loading mechanical equipment for coating of seeds | BBA[a] | Liquid | no PPE | no RPE | 0.343 | 0.0006 | 0.114 |
| | | Solid (WP) | no PPE | no RPE | 0.857 | 0.0700 | 0.292 |
| Mixing and loading granules/treated seeds | PHED | Solid (GR) | no PPE | no RPE | 0.045 | 0.1270 | 0.026 |
| Mixing and loading granules/seeds, Belly Grinder and “Push-type” Rotary Spreaders | | | no PPE | no RPE | Exposure from loading covered in contributing scenario: delivery and dispersion | | |

[a] “Mixing and loading tractor mounted/trailed boom sprayer” used as a surrogate exposure estimate from the BBA model, assuming 10kg substance per day.

**Table S8. Unit exposure parameters for GES2 from PHED (US EPA, 2013) for use with Equations S1 & S2.**

| Contributing Scenario | Unit exposure route ($UE_{derm}$ or $UE_{inhal}$) | Value (mg/kg handled)[a,b] | |
|---|---|---|---|
| | | no PPE | with PPE |
| **PROC 8a - Loading** | | | |
| Loading, Granules | Dermal | 0.01854 | 0.01523 |
| | Hand | 0.004062 | 0.000762 |
| | Inhalation | 0.003753 | - |
| **PROC 8a – Delivery and dispersal** | | | |
| Applicator, Granules by Hand | Dermal | 229.58 | 156.73 |
| | Hand | 79.69 | 7.969 |
| | Inhalation | 1.0375 | - |
| Loader / Applicator, "Push-type" Rotary Spreaders | Dermal | 0.9713 | 0.5298 |
| | Hand | 4.0397 | 0.40397 |
| | Inhalation | 0.02208 | - |
| Loader / Applicator, Belly Grinder | Dermal | 22.075 | 20.530 |
| | Hand | 5.4967 | 3.0684 |
| | Inhalation | 0.1369 | - |
| Applicator, Open Cab Solid Broadcast Spreader | Dermal | 0.02185 | 0.01589 |
| | Hand | 0.006711 | 0.000671 |
| | Inhalation | 0.002649 | - |

[a] Values converted from original reference to metric units. [b] Note in later editions of the Occupational Pesticide Handler Unit Exposure Surrogate Reference Table, some PHED unit exposure values are being replaced.

**Table S9. Parameters for use with Equations S1 & S2 for Table S7 PHED calculations.**

| Variable | Value | Comment |
|---|---|---|
| *AR* | 20 kg.ha-1 (or maximized) | Default application rate (or maximized to a specified RCR) |
| *A* | 20 ha<br>1 ha<br>0.02 ha | Default work area for tractor, mechanical (e.g. belly grinder), and manual (hand) dispersal methods. |
| *UE* | See Table S8 | *UE* is $UE_{inhal}$, $UE_{dermal}$ (or $UE_{hand}$ for local effects). |
| *BW* | 70 kg | REACH worker default |
| *RV* | 10 $m^3$ | REACH worker default, light work |
| PF | 10 & 20 | RPE is optional RMM. No PF is used for gloves with PHED where this is accounted for in the UE. |

**Contributing scenario**: **transfer of treated seeds**

**Table S10. Parameters used with ECETOC TRA (version 3.1) in Table S11 calculations.**

| Input | Transfer of treated seeds |
|---|---|
| Type of setting | industrial |
| Is substance a solid? (yes/no) | Yes |
| Dustiness of solids (high/medium/low) OR VP of volatiles (Pa) at process temperature | high |
| Duration of activity [hours/day] | >4 hours (default) |
| Use of ventilation? | Indoors with LEV |
| Use of respiratory protection and, if so, minimum efficiency? | No |
| Substance in preparation?[a] | >25% |
| Dermal PPE / Gloves | No |
| Consider LEV for dermal exposure? | Yes |

[a] As a reasonable worst-case, it was assumed that the dust arising from bagging treated seeds contains 50% of the co-formulant's initial concentration in the PPP, and the exposure prediction reduced accordingly.

**Table S11. Typical exposure estimate output for GES2 PROC8b transfer of treated seeds.**

| Type of equipment and conditions | Model | Formulation type | PPE | RPE | LEV | Dermal exposure [mg/kg bw/day] | Inhalation Exposure [mg/m³] | Total RCR |
|---|---|---|---|---|---|---|---|---|
| Bagging of treated seeds | ECETOC TRA | Solid | no PPE | no RPE | 95% efficiency | 0.343 | 0.625 | 0.171 |

**Contributing scenario**: **delivery and dispersal of granules and seeds**

**Table S12. Parameters for use with Equations 1 & 2 in Table S13 PHED calculations.**

| | | |
|---|---|---|
| *A* | 0.02 ha | Area treated by hand application |
| *A* | 1 ha | Area treated by mechanical (hand powered) equipment |
| *A* | 20 ha | Area treated by tractor |
| *AR* | 20 kg.ha-1 (or maximized) | Default application rate (or maximized to a specified RCR) |
| *UE* | See Table S7 | *UE* is $UE_{inhal}$, $UE_{dermal}$ (or $UE_{hand}$ for local effects). |
| *BW* | 70 kg | REACH worker default |
| *RV* | 10 $m^3$ | REACH worker default, light work |
| PF | 10 & 20 | RPE is optional RMM. When gloves are assigned to a task, this is accounted for by using the *UE* for gloved hands, or *PF*=10 where measured data are not available. |

**Table S13. Typical exposure estimate output for GES2 PROC8a, delivery and dispersal of granules and treated seeds.**

| Type of equipment and conditions | Model | Formulation type | PPE | RPE | Dermal exposure [mg/kg bw/day] | Inhalation Exposure [mg/m³] | Total RCR |
|---|---|---|---|---|---|---|---|
| Applicator, Granules by Hand | PHED | Solid (GR) | no PPE | no RPE | 0.555 | 0.018 | 0.187 |
| Loader / Applicator, “Push-type” Rotary Spreaders | | | no PPE | no RPE | 0.117 | 0.019 | 0.041 |
| Loader / Applicator, Belly Grinder | | | no PPE | no RPE | **2.668** | **0.116** | **0.900** |
| Applicator, Open Cab Solid Broadcast Spreader | | | no PPE | no RPE | 0.016 | 0.090 | 0.014 |

## Combined exposure for seed and granular application

**Table S14. Typical combined task exposure estimate for GES2 treated seed and granules, and used for maximum use rate calculations.**

| Contributing Scenarios | Use rate | | Dermal exposure [mg/kg bw/day] | Inhalation Exposure [mg/m³] | PPE | RPE | LEV | Dermal RCR | Inhalation RCR | Total RCR |
|---|---|---|---|---|---|---|---|---|---|---|
| | [kg/ha] | [kg/d] | | | | | | | | |
| **Seed treatment** | | | | | | | | | | |
| PROC 8a: Mixing & loading WP formulation into batch treater | **n.a.** | **10.00** | 0.857 | 0.070 | no PPE | no RPE | | 0.286 | 0.006 | 0.292 |
| PROC 8b: Bagging of treated seeds | | | 0.343 | 0.625 | no PPE | no RPE | 95% efficiency | 0.114 | 0.057 | 0.171 |
| **PROC 8a+8b** | | | **1.200** | **0.695** | | | | **0.400** | **0.063** | **0.463** |
| **Dispersion of granules or treated seeds, tractor** | | | | | | | | | | |
| PROC 8a: Mixing and loading granules (including treated seeds) | **8.5** | **169.2** | 0.045 | 0.127 | no PPE | no RPE | | 0.015 | 0.012 | 0.026 |
| PROC 8a: Applicator, open cab solid broadcast spreader | | | 0.016 | 0.090 | no PPE | no RPE | | 0.005 | 0.008 | 0.014 |
| **PROC 8a+8a** | | | **0.061** | **0.217** | | | | **0.020** | **0.020** | **0.040** |
| **Dispersion of granules or treated seeds, mechanical** | | | | | | | | | | |
| PROC 8a: Spreading of granules or treated seeds using belly grinder, including loading of equipment | **8.5** | **8.5** | **2.668** | **0.116** | no PPE | no RPE | | **0.889** | **0.011** | **0.900** |

## GES3 AND GES4 FOR CONSUMER USES

The OWB applicability domain for the GES3 exposure predictions covers the transfer (and inherent diluting and mixing) of solid and liquid PPPs which occurs during loading of hand-held spray equipment, and the spray application of PPPs using hand-held spray equipment on high-level targets. The applicability domain for the GES4 exposure predictions covers the manual spreading by hand, spoon, cup, push-type rotary spreader, or belly grinder, and for granular PPPs or treated seeds on residential lawns, turf, gardens and trees. Further details on these models are given below.

Example calculations by ECPA OWB v3.3 are provided in Tables S15, S16, and S17. The same input parameters given in the footnote to Table 4 have been used for consistency.

**Table S15. Typical exposure estimate output for GES3, spray application of PPPs by consumers.**

| Type of equipment and conditions | Model | Formulation type | PPE[a] | RPE[a] | Dermal exposure [mg/kg bw/day] | Inhalation Exposure [mg/m³] | Total RCR |
|---|---|---|---|---|---|---|---|
| Mixing and loading hand-held sprayer, outdoors | BBA | **Liquid** | **-** | **-** | **0.935** | **0.0006** | **0.6238** |
| | | Granule (WG) | - | - | 0.373 | 0.0004 | 0.2486 |
| | | Powder (WP) | - | - | 0.373 | 0.0100 | 0.2523 |
| Hand-held spraying, high-level target, outdoors | | Liquid | - | - | 0.411 | 0.0054 | 0.2762 |

[a] PPE and RPE is not used for consumer risk assessments.

**Table S16. Typical combined task exposure estimate for GES3 PC27, and used for maximum use rate calculations.**

| Sub-activities | Use rate | | Dermal exposure [mg/kg bw/day] | Inhalation Exposure [mg/m³] | PPE[a] | RPE[a] | Dermal RCR | Inhalation RCR | Total RCR |
|---|---|---|---|---|---|---|---|---|---|
| | [kg/ha] | [g/d] | | | | | | | |
| Task 1: Mixing & loading liquid formulation into knapsack sprayer | 6.52 | 130 | 0.9354 | 0.0006 | - | - | 0.624 | 0.0002 | 0.624 |
| Task 2: Hand-held spraying, outdoors | | | 0.411 | 0.0054 | - | - | 0.274 | 0.0021 | 0.276 |
| Task 1 + 2 | | | 1.347 | 0.006 | - | - | 0.898 | 0.0023 | 0.900 |

[a] PPE and RPE is not used for consumer risk assessments.

**Table S17. Typical exposure estimate output for GES4 PC27, dispersal of granules or treated seeds by consumers.**

| Type of equipment and conditions | Model | Formulation type | PPE[b] | RPE[b] | Dermal exposure [mg/kg bw/day] | Inhalation Exposure [mg/m³] | Total RCR |
|---|---|---|---|---|---|---|---|
| "Push-type" Spreaders | SOPREA[a] | Solid (GR) | - | - | 0.003 | 0.0000 | 0.002 |
| Belly grinders | | | **-** | **-** | **1.350** | **0.0004** | **0.900** |
| Hand dispersal, spoon | | | - | - | 0.023 | 0.0010 | 0.016 |
| Hand dispersal, cup | | | - | - | 0.000 | 0.0001 | 0.000 |
| Hand dispersal | | | - | - | 0.600 | 0.0043 | 0.402 |

[a] SOPREA: US EPA SOP for Residential Exposure Assessments, Feb. 2012. [b] PPE and RPE is not used for consumer risk assessments.

## BENCHMARKING GES1 AND GES2

Benchmarking was carried out against ECETOC TRA (version 3.1), which is a typical tier one exposure model used in the risk assessment of substances for REACH. Two hypothetical substances were considered, one a solid with a vapour pressure of 0.001 Pa, and the second a liquid with vapour pressure of 0.001 Pa. The change in physical state at the same vapour pressure was selected to highlight the influence of fugacity in the different exposure models.

Model inputs were chosen to match as closely as possible the worst-case task identified by the ECPA OWB tool (see Table 2, 3, 4 and Tables S9, S11, S13-17). The co-formulant concentration in concentrated PPPs (WP) and diluted spray solutions is assumed to be 100% and 1%, respectively.

In the REACH models the durations of the mixing and loading tasks are assumed to be 60 minutes per shift. The spray applications are assumed to have a duration of 360 minutes. In the PPP models the exposure is linked to the work area, rather than duration. These are assumed to be full shift values.

### Model Inputs for Benchmarking

### ECPA OWB

The ECPA OWB (version 3.3) was used to calculate exposures for GES1 and GES2.

Substance 1: physical state = solid, vapour pressure = 0.001 Pa.

Substance 2: physical state = liquid, vapour pressure = 0.001 Pa.

Worst-case exposures were taken from tabs “PPP GES1” tables PROC 8a and PROC 11, and “PPP GES2” tables PROC 8a, PROC 8b and PROC 8a. The default application rates of

1 kg/ha and 20 kg/ha were used. No PPE or RPE was specified for any contributing scenario or activity.

**BBA**

The benchmarking calculations for GES1 were carried out using the PSD excel implementation of the BBA model (German_Model_PSD1.xls). Dermal exposure predicted by the model was normalised to worker body weight using 70 kg. Inhalation exposure predicted by the model was normalised using the worker respiratory volume for light work = 10 m³/8 h. The worst-case tasks had previously been identified during the course of the ECPA OWB development.

**Table S18. GES1 input parameters to the BBA model for a solid substance with vapour pressure = 0.001 Pa.**

| | **Contributing scenario** | | | |
|---|---|---|---|---|
| | **Tractor-mounted** | | **Hand-held** | |
| **Input parameter** | Mixing and loading | Spray applications | Mixing and loading | Spray applications |
| Use descriptor | PROC8a | PROC11 | PROC8a | PROC11 |
| Active substance concentration | 1000 g/kg | 1000 g/kg | 1000 g/kg | 1000 g/kg |
| Dermal absorption from product | 100% | 100% | 100% | 100% |
| Dermal absorption from spray | 100% | 100% | 100% | 100% |
| RPE | none | none | none | none |
| PPE | none | none | none | none |
| Dose | 1 kg product/ha | 1 kg product/ha | 1 kg product/ha | 1 kg product/ha |
| Work rate / day (fixed) | 20 ha | 20 ha | 1 ha | 1 ha |
| Application method | Tractor-mounted/trailed boom sprayer: hydaulic nozzles | Tractor-mounted/trailed broadcast air-assisted spraying | Hand-held sprayer: hydaulic nozzles. Outdoor, high-level target | Hand-held sprayer: hydaulic nozzles. Outdoor, high-level target |
| Formulation type | WP | Liquid | Liquid | Liquid |

For Substance 2, a liquid with vapour pressure = 0.001 Pa, the following changes were made PROC 8a: application method = “Tractor-mounted/trailed boom sprayer: hydaulic nozzles”, Formulation type = liquid, on the basis that WP and WG are not relevant formulation types for a liquid substance. No change was made for PROC 8a “Hand-held sprayer: hydaulic nozzles. Outdoor, high-level target”, because Formulation type = Liquid was the worst case. Similarly for PROC 11, because the diluted spray formulation is a liquid, no change to inputs was required.

Although the ECPA OWB uses a BBA model prediction for a GES2 contributing scenario, because this is an analogous task requiring further justification, it is considered to be out-of-scope of the standard use of the model and not included here.

**PHED**

The benchmarking calculations for PHED were carried out using Equations 1 & 2 to predict dermal and inhalation exposure with the following values:

**Table S19. Parameters used for GES2 PROC8a, using "Mixing and loading of granules".**

| Variable | Value | Comment |
|---|---|---|
| *AR* | 20 kg/ha | Default application rate |
| *A* | 20 ha | Default work area for tractor |
| $UE_{derm}$[a] | 8.4 µg/lb | Mixing / Loading Granules, single layer no gloves. |
| $UE_{inhal}$[a] | 1.7 µg/lb | Mixing / Loading Granules, no respirator. |
| *BW* | 70 kg | REACH worker default |
| *RV* | 10 $m^3$ | REACH worker default, light work |
| PF | 1 | No PPE or RPE was considered in benchmarking exercise. |
| | 0.00221 | Conversion µg/lb to mg/kg |

[a] Occupational pesticide handler unit exposure surrogate reference table, March 2013

**Table S20. parameters used for GES2 PROC8a, using "Loader / Applicator, Belly Grinder".**

| Variable | Value | Comment |
|---|---|---|
| *AR* | 20 kg/ha | Default application rate |
| *A* | 1 ha | Default work area for tractor |
| $UE_{derm}$[a] | 10000 µg/lb | Loader / Applicator, Belly Grinder, single layer no gloves. |
| $UE_{inhal}$[a] | 62 µg/lb | Loader / Applicator, Belly Grinder, no respirator. |
| *BW* | 70 kg | REACH worker default |
| *RV* | 10 $m^3$ | REACH worker default, light work |
| PF | 1 | No PPE or RPE was considered in benchmarking exercise. |
| | 0.00221 | Conversion µg/lb to mg/kg |

[a] Occupational pesticide handler unit exposure surrogate reference table, March 2013

**EFSA**

The EFSA AOEM model was used to estimate exposures for the ECPA GES1 and GES2 contributing scenarios. In the absence of a detailed assessment of the input parameters, their dependencies, and distributions, exposure estimates for the contributing scenarios were obtained by selecting the inputs most closely matching the worst-case use patterns identified in the ECPA OWB Tables 2 and 3, and Tables S9, S10, and S11. As a result, the selected

input parameters are not necessarily the worst-case combinations. This highlights the practical difficulty in using such tools for screening level assessments, and the inter-assessor variability that is anticipated to arise in the absence of a standardised framework (e.g. Money et al., 2016).

Dermal exposure was assumed to be the sum of the AOEM estimate for Hand, Body and Head (given in the Operator Outdoor Spray AOEM worksheet, Mixing & Loading and Application intermediate values box). REACH default values for respiratory volume and worker body weight were used for conversion to unit exposure values. 75$^{th}$ percentile exposure predictions were used in line with the EFSA model guidance. Exposure estimates for Substance 1 assumed a solid substance with VP = low in a Wettable Powder formulation. Substance 2 assumed a liquid substance with VP = low, in a Soluble Concentrate formulation.

**Table S21. GES1 input parameters to the EFSA AOEM tool (version 30$^{th}$ March 2015).**

| | **Contributing scenario** | | | |
|---|---|---|---|---|
| | **Tractor-mounted** | | **Handheld** | |
| **Input parameter** | Mixing and loading | Spray applications | Mixing and loading | Spray applications |
| Use descriptor | PROC8a | PROC11 | PROC8a | PROC11 |
| Area treated | 50 ha/day | 10 ha/day | 4 ha/day | 4 ha/day |
| Crop type | cereal | citrus fruit | citrus fruit | citrus fruit |
| Formulation type | Wettable powder (solid) / Sol. Conc. (liquid) | Wettable powder (solid) / Sol. Conc. (liquid) | Wettable powder (solid) / Sol. Conc. (liquid) | Wettable powder (solid) / Sol. Conc. (liquid) |
| Maximum application rate of active substance | 1kg/ha | 1kg/ha | 1kg/ha | 1kg/ha |
| Vapour pressure of active substance | Low | Low | Low | Low |
| Indoor or Outdoor application | Outdoor | Outdoor | Outdoor | Outdoor |
| Application method | Downward spraying | Upward spraying | Upward spraying | Upward spraying |
| Application equipment | Vehicle-mounted | Vehicle-mounted | Manual-Hand held | Manual-Hand held |
| Buffer strip | 2-3 m | 2-3 m | 2-3 m | 2-3 m |
| Number of applications | 1 | 1 | 1 | 1 |
| Interval between multiple applications | 365 | 365 | 365 | 365 |
| Season (upward spraying orchards only) | Not relevant | Not relevant | Not relevant | Not relevant |

### Table S22. GES2 input parameters to the EFSA AOEM tool (version 30th March 2015).

| Input parameter | Contributing scenario | |
|---|---|---|
| Contributing scenario | **Mixing and loading** | **Dispersal of granules and seeds** |
| Use descriptor | PROC8a | PROC8a |
| Area treated | 50 ha/day | 1 ha/day |
| Crop type | cereals | cereals |
| Formulation type | Granules | Granules |
| Maximum application rate of active substance | 20 kg/ha | 20 kg/ha |
| Vapour pressure of active substance | Low | Low |
| Indoor or Outdoor application | Outdoor | Outdoor |
| Application method | Broadcast application of granules | Manual application of granules |
| Application equipment | Vehicle-mounted | Manual |
| Buffer strip | 2-3 m | 2-3 m |
| Number of applications | 1 | 1 |
| Interval between multiple applications | 365 | 365 |
| Season (upward spraying orchards only) | Not relevant | Not relevant |

## ECETOC TRA

For GES1 the substance was assumed to be solid with vapour pressure = 0.001 Pa, or a liquid with vapour pressure = 0.001 Pa, with molecular weight = 100 g/mol.

### Table S23. GES1 input parameters for ECETOC TRA (version 3.1), for a solid with vapour pressure = 0.001 Pa.

| Input | Contributing scenarios | | | |
|---|---|---|---|---|
| | **Mixing and loading** | | **Spray application** | |
| Activity | Tractor | Hand-held | Tractor | Hand-held |
| Process category (PROC) | PROC8a | PROC8a | PROC11 | PROC11 |
| Type of setting | professional | professional | professional | professional |
| Is substance a solid? (yes/no) | Yes | Yes | No* | No* |
| Dustiness of solids (high/medium/low) OR VP of volatiles (Pa) at process temperature | high | high | 0.001 | 0.001 |
| Duration of activity [hours/day] | 15 min – 1 hour | 15 min – 1 hour | >4 hours (default) | >4 hours (default) |
| Use of ventilation? | outdoors | outdoors | outdoors | outdoors |
| Use of respiratory protection and, if so, minimum efficiency? | No | No | No | No |
| Substance in preparation? | >25% | >25% | <1% | <1% |
| Dermal PPE / Gloves | No | No | No | No |
| Consider LEV for dermal exposure? | No | No | No | No |

* Marquart et al. (2017) recently validated that ECETOC TRA can make useful dermal exposure predictions for a solid dispersed/dissolved in a liquid, provided that it is treated as a negligible vapour-pressure liquid. It should be noted that ECETOC TRA inhalation exposure predictions are for vapours and not aerosols, and thus these exposure estimates lie outside the applicability domain.

**Table S24. GES1 input parameters for ECETOC TRA (version 3.1), for a liquid with vapour pressure = 0.001 Pa.**

| Input | Contributing scenarios | | | |
|---|---|---|---|---|
| | Mixing and loading | | Spray application | |
| Activity | Tractor | Hand-held | Tractor | Hand-held |
| Process category (PROC) | PROC8a | PROC8a | PROC11 | PROC11 |
| Type of setting | professional | professional | professional | professional |
| Is substance a solid? (yes/no) | no | no | no | no |
| Dustiness of solids (high/medium/low) OR VP of volatiles (Pa) at process temperature | 0.001 | 0.001 | 0.001 | 0.001 |
| Duration of activity [hours/day] | 15 min – 1 hour | 15 min – 1 hour | >4 hours (default) | >4 hours (default) |
| Use of ventilation? | outdoors | outdoors | outdoors | outdoors |
| Use of respiratory protection and, if so, minimum efficiency? | No | No | No | No |
| Substance in preparation? | >25% | >25% | <1% | <1% |
| Dermal PPE / Gloves | No | No | No | No |
| Consider LEV for dermal exposure? | No | No | No | No |

For GES2 the substance was assumed to be solid with vapour pressure = 0.001 Pa, with molecular weight = 100 g/mol.

**Table S25. GES2 input parameters for ECETOC TRA (version 3.1).**

| Input | Contributing scenarios | | |
|---|---|---|---|
| | Mixing and loading | Transfer of treated seeds | Dispersal of granules and seeds |
| Process category (PROC) | PROC8a | PROC8b | PROC8a |
| Type of setting | professional | industrial | professional |
| Is substance a solid? (yes/no) | Yes[a] | Yes | Yes |
| Dustiness of solids (high/medium/low) OR VP of volatiles (Pa) at process temperature | high | high | high |
| Duration of activity [hours/day] | 15 min – 1 hour | >4 hours (default) | >4 hours (default) |
| Use of ventilation? | outdoors | Indoors with LEV | outdoors |
| Use of respiratory protection and, if so, minimum efficiency? | No | No | No |
| Substance in preparation? | >25% | >25% | >25% |
| Dermal PPE / Gloves | No | No | No |
| Consider LEV for dermal exposure? | No | Yes | No |

[a] Solid, vapour pressure = 0.001 Pa.

## Collated Benchmarking Results

**Table S26. Collated results of the exposure model benchmarking calculations for GES1 and GES2 contributing scenarios. Values are for: powder VP=0.001 Pa / liquid VP=0.001 Pa, at constant 1kg/ha use rate.**

| | Task | Route | ECPA OWB | Pesticide models | | | REACH tier one models |
|---|---|---|---|---|---|---|---|
| | | | | BBA[a,b,d] | PHED[a,e] | AOEM[a,b,f] | ECETOC TRA[c] |
| GES 1[g] | PROC 8a - mixing and loading (tractor-mounted)[i,j] | dermal (mg/kg bw/day) | 1.714 / 0.686 | 1.714 / 0.686 | - | 25.6 / 2.25 | 13.7 / 13.7 |
| | | inhalation (mg/m³) | 0.140 / 0.0012 | 0.140 / 0.0012 | - | 0.696 / 0.0012 | 7.0 / 0.058 |
| | PROC 8a - mixing and loading (hand-held)[i,j] | dermal (mg/kg bw/day) | 2.929 / 2.929 | 2.929 / 2.929 | - | 4.15 / 0.340 | 13.7 / 13.7 |
| | | inhalation (mg/m³) | 0.005 / 0.005 | 0.005 / 0.005 | - | 0.328 / 0.0006 | 7.0 / 0.058 |
| | PROC 11 - tractor mounted spraying[k,l] | dermal (mg/kg bw/day) | 1.314 / 1.314 | 1.314 / 1.314 | - | 1.70 / 1.70 | 10.7 / 10.7 |
| | | inhalation (mg/m³) | 0.0144 / 0.0144 | 0.0144 / 0.0144 | - | 0.023 / 0.023 | 29.2 / 29.2 |
| | PROC 11 - hand held spraying[k,l] | dermal (mg/kg bw/day) | 0.577 / 0.577 | 0.577 / 0.577 | - | 1.22 / 1.22 | 10.7 / 10.7 |
| | | inhalation (mg/m³) | 0.030 / 0.030 | 0.030 / 0.030 | - | 0.0255 / 0.0255 | 29.2 / 29.2 |
| GES 2[h] | PROC 8a - mixing and loading[i,j] | dermal (mg/kg bw/day) | 0.857 / 0.343 | - | 0.106 | 0.025 | 13.7 |
| | | inhalation (mg/m³) | 0.070 / 0.0006 | - | 0.150 | 0.002 | 7.0 |
| | PROC 8b - bagging treated seeds[k,j] | dermal (mg/kg bw/day) | 0.343 / 0.343 | - | - | - | 0.686 |
| | | inhalation (mg/m³) | 0.625 / 0.625 | - | - | - | 1.25 |
| | PROC 8a - dispersion of granular PPPs or treated seeds[k,l] | dermal (mg/kg bw/day) | 6.307 / 6.307 | - | 6.307 | 2.78 | 13.7 |
| | | inhalation (mg/m³) | 0.274 / 0.274 | - | 0.274 | 0.0009 | 35.0 |

[a] Values normalised using REACH default 70 kg worker and 10 $m^3$ respiratory volume (ECHA, 2012). [b] Model predicts spray exposure. [c] Model predicts vapour phase exposure. [d] geometric mean. [e] "best fit". [f] 75$^{th}$ percentile. [g] 1 kg/ha application rate. [h] 20 kg/ha application rate. [i] 60 min task duration. [j] 100% co-formulant concentration. [k] 360 min task duration, or full shift. [l] 1% co-formulant concentration.

## References


Brouwer DH, de Haan M, Van Hemmen JJ (2000). Modeling re-entry exposure estimates. Application techniques and rates. In: Honeycutt RC, Day EW Jr (eds.), Worker Exposure to Agrochemicals. ACS Symposium Series. CRC Lewis Publishers, Baton Rouge, USA, pp 119-138.

Delmaar JE, Park MVDZ, van Engelen JGM (2005) ConsExpo 4.0 – Consumer exposure and uptake models program manual. RIVM report 320104004/2005. RIVM Bilthoven, the Netherlands

ECHA (2012) Guidance on information requirements and chemical safety assessment. Chapter R.8: Characterisation of dose [concentration]-response for human health. Version 2.1, Nov 2012. Available from: URL:

http://echa.europa.eu/documents/10162/13632/information_requirements_r8_en.pdf (accessed 19 Oct 2015)

van Hemmen JJ, Chester G, Hamey P, Kangas J, Kirknel E, Maasfeld W, Perkins J, Phillips J, Schulze-Rosario C (2002) Post-application exposure of workers to pesticides in agriculture. EUROPOEM II PROJECT FAIR3-CT96-1406. Draft Report of the Re-entry Working Group, December 2002

Hoernicke E, Nolting H-G, Westphal D (1998), Hinweise in der Gebrauchsanleitung zum Schutz von Personen bei Nachfolgearbeiten in mit Pflanzenschutzmitteln behandelten Kulturen (worker re-entry), Nachrichtenbl Deut Pflanzenschutzd; 50: 267-269

Krebs B, Maasfeld W, Schrader J, Wolf R, Hoernicke E, Nolting HG, Backhaus GF, Westphal D (2000) Uniform Principles for Safeguarding the Health of Workers Re-entering Crop Growing Areas after Application of Plant Protection Products. Nachrichtenbl Deut Pflanzenschutzd; 52: 5-9

Lundehn J-R, Westphal D, Kieczak H, Krebs B, Löcher-Boltz S, Maasfeld W and Pick ED (1992) Uniform principles for safeguarding the health of applicators of plant protection products. Mitteilungen aus der Biologischen Bundesanstalt für Land- und Forstwirtschaft, Heft 277, Berlin, Germany.

Martin S, Westphal D, Erdtmann-Vourliotis M, Dechet F, Schulze-Rosario C, Stauber F, Wicke H, Chester G (2008) Guidance for Exposure and Risk Evaluation for Bystanders and Residents exposed to Plant Protection Products during and after Application. J Verbr Lebensm; 3: 272 – 281

Money A, Robinson C, Agius R, de Vocht F (2016) Wishful Thinking? Inside the Black Box of Exposure Assessment. Ann Occup Hyg; 60(4): 421–431.

Rautmann D, Streloke M, Winkler R (2001): New basic drift values in the authorization procedure for plant protection products. Mitteilungen aus der Biologischen Bundesanstalt für Land- und Forstwirtschaft; 383: 133-141.

US EPA (2012) Standard Operating Procedures (SOPs) for Residential Exposure Assessments, February 2012, page 3-3, available at http://www.epa.gov/opp00001/science/EPA-OPP-HED_Residential%20SOPS_Feb2012.pdf (accessed 8 June 2012))

US EPA (2013) Occupational Pesticide Handler Unit Exposure Surrogate Reference Table, March 2013.
